# Histochemical Study of the Progenetic Trematode *Alloglossidium renale*

**DOI:** 10.1155/2014/245769

**Published:** 2014-09-10

**Authors:** Craig A. Schimmer, Stephen C. Landers

**Affiliations:** Department of Biological and Environmental Sciences, Troy University, Troy, AL 36082, USA

## Abstract

A histochemical study of the progenetic trematode *Alloglossidium renale* has demonstrated the absence of any secreted material between the adult worm and the host (freshwater shrimp) antennal gland tubules. Host tissue is affected only by the compression, abrasion, and ingestion by the parasite, and host tubule cells near the worm have the same staining patterns as those distant from the parasite. The trematode sometimes dies within the host, leaving a necrotic mass whose histochemical staining differs significantly from the living organism. In the necrotic mass, the only recognizable features were the ova and the vitellarium, which atrophied and resulted in tyrosine-positive staining within the mass. A melanin reaction was not observed in the host using a specialized ferro-ferricyanide stain. The only apparent host response to infection was a layer of damaged squamous host cells adhering to the necrotic worm. The results confirm benign host-parasite effects and a highly evolved relationship between the host and parasite, perhaps bordering on commensalism.

## 1. Introduction

*Alloglossidium renale* is a progenetic trematode that matures within the antennal glands of the grass shrimps* Palaemonetes kadiakensis* and* P. paludosus* [1, 2]. Many articles have focused on the ecology of* A. renale* or the phylogenetic relationship of this trematode to others [2–7] but there has been only a single examination of the* in situ* anatomy of the host-parasite relationship [8].

In south Alabama,* A. renale* infects the antennal glands of the freshwater shrimp* P. kadiakensis* [8], which is a common species of shrimp found throughout south Alabama's rivers and streams [9]. The known life cycle of* A. renale* begins with the cercariae stage infecting* P. kadiakensis*. The cercariae bore into the host and develop within the antennal gland, where they mature to a progenetic metacercaria stage [6, 10]. The worm reaches sexual maturity in six weeks [10] at which time it produces large numbers of eggs which are released from the shrimp's excretory pore. Earlier it was reported that a host reaction was not observable with sectioned material from plastic-embedded specimens [8]. This current study has used a variety of histochemical stains to examine the host tissue, mature parasite, and necrotic parasite (after the death of the trematode) and reports the changes that occur during the host-parasite relationship upon the senescence of the worm.

## 2. Materials and Methods

### 2.1. Collection Site

*Palaemonetes kadiakensis* was collected from Olustee Creek in Pike County, Alabama (31°56′38′′N, 86°7′6′′W). This site is a wadeable road-side location, utilized in previous studies for grass shrimp infected with* A. renale* [8]. Host organisms were obtained by dip-netting along the sides of the creek. Hosts were transported to Troy University in water collected from the site. Shrimp were identified using Pennak [11].

### 2.2. Laboratory Identification of Infections

Shrimp were examined for infections using a dissection microscope.* Alloglossidium renale* is located in the antennary glands (antennal gland or kidney) of the host, which are located posterior to the antennal scales and anterior to the gill chamber on each side of the cephalothorax.* Palaemonetes kadiakensis* has a translucent exoskeleton, making external diagnosis possible. In an earlier study, the prevalence of infection at Olustee Creek was approximately 13%, with shrimp often hosting multiple trematodes in one or both antennal glands [8]. Voucher specimens of* A. renale* from infected shrimp at this location were deposited in the US National Parasite Collection in Beltsville, MD (USNPC 101574), during that earlier study by this lab [8]. Necrotic parasites were identified as dark masses at the base of the antennal scales without a distinct uterus.

### 2.3. Paraffin Sections

The cephalothorax of an infected host was removed and fixed using Bouin's fixative or 10% buffered formalin for 24 hours depending on the specific histochemical test to be used. Fish (*Pristipomoides aquilonaris*) intestine was fixed for select control experiments. The tissue was then dehydrated using 50%, 70%, 95%, and two 100% isopropanol solutions for 24 hours each, transferred to Safeclear solvent, a 60°C Safeclear-paraffin solution (1 : 1 ratio), and two 60°C paraffin solutions for 24 hours each. The tissue was then cast in paraffin blocks, sectioned at a 10 *μ*m thickness using a rotary microtome (American Optical), and affixed onto subbed slides.

### 2.4. Staining and Photography

Paraffin was removed from the tissue with Safeclear (three changes, 30 min each). The tissue was then hydrated in an isopropanol: distilled water series before staining (Table 1). In addition to examining the basic anatomy of the host-parasite relationship with Harris hematoxylin [HH], a variety of stains were selected to test for acidic mucosubstances (alcian blue pH 1.0 [AB1], alcian blue pH 2.5 [AB2.5], toluidine blue [TB]), neutral sugars (periodic acid-Schiff [PAS]), DNA (Feulgen nuclear reaction or HCL-Schiff [FL]), proteins (ninhydrin-Schiff [NS], Ward's protein stain [WPS]), lipids (osmium tetroxide [OS]), and tyrosine/melanin (ferro-ferricyanide [FF]). Staining protocols followed various sources [12, 13]. Counter stains were not used. Permanent preparations were made using standard dehydration, clearing, and mounting techniques [14]. Plastic sections of tissue fixed in osmium tetroxide were examined from material prepared for a previous study [8]. Photographs were made with a Nikon DXM 1200 digital camera mounted on a Nikon E600 light microscope. Images were adjusted for brightness, contrast, and gamma using Adobe Photoshop Elements to aid in the interpretation of the staining. Histochemical results were graded using this semiquantitative scale: +++: intense stain, ++: moderate stain, +: light stain, and −: no stain.

## 3. Results

### 3.1. Summary of* Alloglossidium renale *Histochemistry (Figures 1 and 2, Table 2)

**Figure 1:**
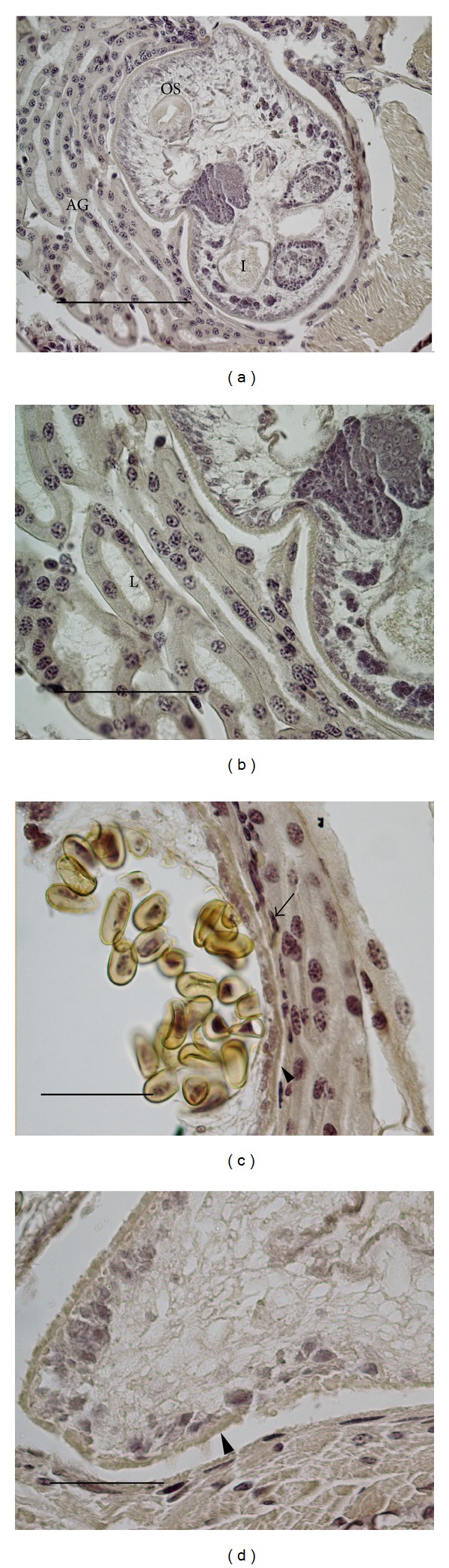
Harris hematoxylin staining of mature* Alloglossidium renale* within the host. (a) Overview of the parasite. The host antennal gland tubules are on the left side of the image and host striated muscle is on the lower right. (b) An enlargement of (a). (c) Area showing the compression of host antennal gland tubules with compacted nuclei (arrow). The parasite integument has an outer anucleate syncytium (arrowhead). (d) The parasite has consumed the antennal gland. Host muscle is located in the lower part of the image. The parasite integument is indicated (arrowhead). AG: antennal gland; I: intestine; L: Lumen of antennal gland tubule; and OS: oral sucker. (a) Bar = 200 *μ*m, (b) bar = 100 *μ*m, and (c)–(d) bars = 50 *μ*m.

Harris hematoxylin provided a basis for interpreting the results of the histochemical staining (Figure 1). The outer syncytium of the worm lacked nuclei, which were below the surface. No trace of a metacercarial cyst wall was observed between the host antennal gland tubules and the integument of the parasite. The parenchyma of the parasite was loose and stained lightly. Other identifiable structures included the vitellarium, suckers, gonads, ova, and intestine.

**Figure 2:**
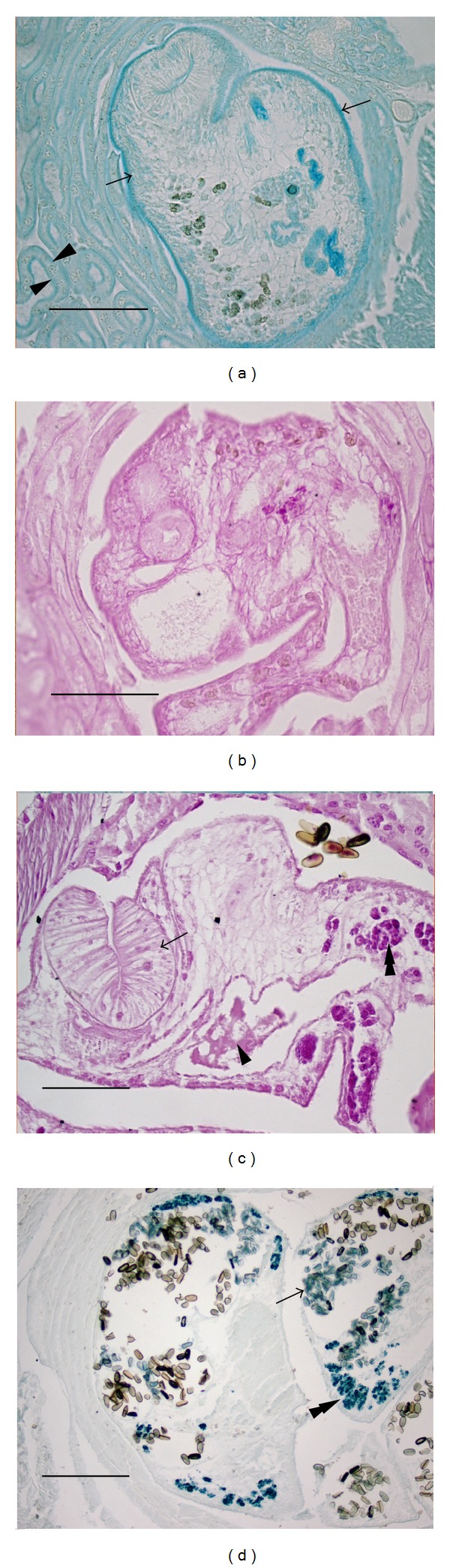
Histochemical staining of healthy* A. renale* within the host. (a) Alcian blue pH 2.5 staining for acidic mucosubstances. The tegument of the worm stains strongly (arrows) as do the apical and basal borders of the cuboidal antennal gland tubule cells (arrowheads). No metacercarial wall is present. (b) Periodic acid-Schiff staining for neutral carbohydrates reveals uniform staining of* A. renale* and no metacercarial wall. (c) Ninhydrin-Schiff staining for amino acids reveals light staining of* A. renale* parenchyma and no metacercarial wall. Arrow: oral sucker; arrowhead: intestinal contents; and double arrowhead: vitellarium. (d) Ferro-ferricyanide staining reveals tyrosine-based material within the vitellarium (double arrowhead) and egg shells (arrow) but no melanin reaction from the host tissue. (a)–(c) Bars = 100 *μ*m. (d) Bar = 200 *μ*m.

Carbohydrate staining with AB1, AB2.5, PAS, and TB provided consistent results. Acidic mucosubstances stained with the AB stains were present mostly in the outer tegument of the worm, the intestinal lining, and reproductive tubules (Figure 2(a)). The parenchyma was negative for acidic sugars with the AB stains. Alcian blue 2.5 also stained mucoid material outside of the body but trapped within the cup of the suckers. Toluidine blue revealed an outer pink or light blue tegument underlain by a darker layer, indicating negatively charged material in the syncytium. The PAS reaction revealed neutral sugars throughout the worm (Figure 2(b)), with the outer tegumental syncytium staining being lighter than the subsurface region.

Protein staining with NS and WPS was used to examine whether any external secretions could be detected as a remnant or poorly formed metacercarial cyst wall. No such external secretion was detected, and the staining for proteins was consistent for most structures within the parasite (Figure 2(c)). Parenchymal staining was less intense for NS than the PAS stain, though the vitellarium stained intensely with NS.

Nuclear staining using FL confirmed the results of the HH procedure in that the nuclei of the integument were recessed below the outer syncytium. Nuclei were scattered throughout the loose parenchyma and were found in typical locations throughout the rest of the worm. Oil droplets were occasionally observed in plastic sections of worms fixed for EM using osmium tetroxide.

Melanin or melanin components such as tyrosine were detected very strongly in the vitellarium using the FF stain (Figure 2(d)). Additionally, the FF procedure stained the egg shell material. No other area of the parasite stained positively.

### 3.2. Summary of Host Antennal Gland Tissue Histochemistry (Table 3, Figures 1 and 2)

Harris hematoxylin was used as a stain for* P. kadiakensis* general antennal gland structure (Figure 1). The microvilli at the apical end of the antennal gland tubule cells and the cytoplasm of the tubule cells stained lightly, with the basal border staining slightly darker (Figure 1(b)). DNA within the nuclei of the tubule cells stained darkly. This staining characterized healthy nuclei as round structures with clear nucleoplasm surrounding islands of DNA. The lumenal contents of the tubules stained lightly. Damaged tubule cells were compacted and not cuboidal. They transitioned to squamous cells near the parasite (Figure 1(c)), likely due to the growth of the worm and compression of the host kidney tubules. In some infections, most of the antennal gland was consumed by the parasite, and the worm abutted directly against the muscle or was separated by a thin squamous tubule cell layer (Figure 1(d)). Hematoxylin staining revealed that nuclei of damaged, tubule cells were flattened and dense, without large areas of light nucleoplasm. The identity of the flattened cell nuclei was confirmed with the FL DNA stain.

**Figure 3:**
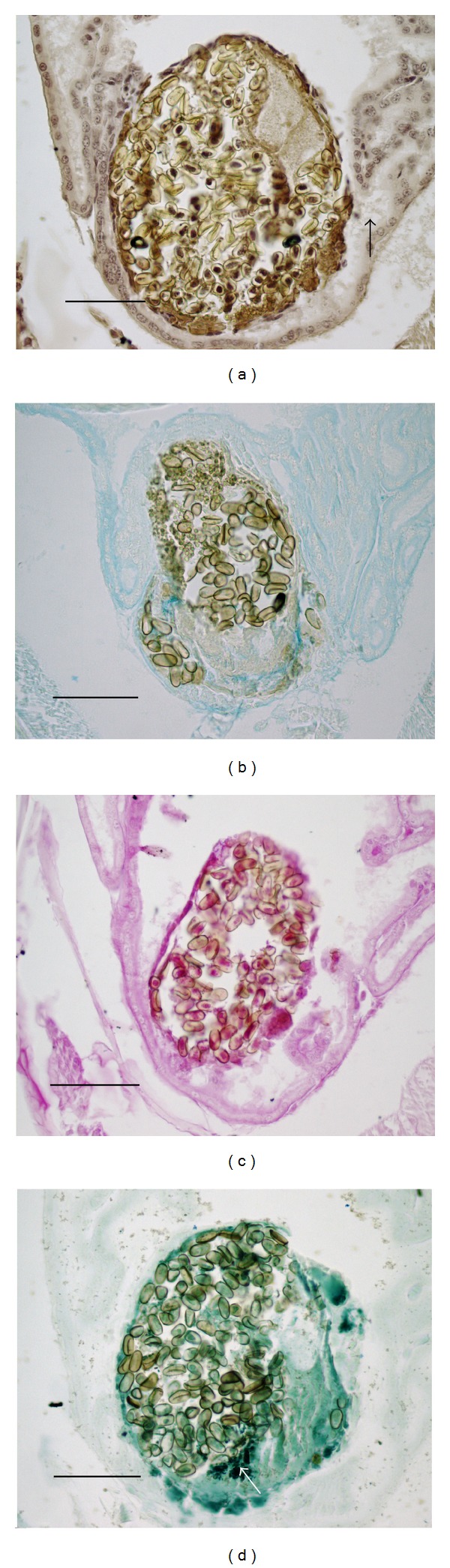
Histochemical staining of necrotic* A. renale* within the host. (a) Harris hematoxylin staining reveals an unrecognizable mass of material with ova. Host antennal gland tubule lumina contain lightly stained material (arrow). (b) Alcian blue pH. 2.5 staining reveals a change in the staining pattern compared to healthy trematodes, with no distinct integument. Staining of the host tissue reveals the same results as shown with healthy parasites. (c) Periodic acid-Schiff staining for neutral sugars reveals strongly stained areas of material on the necrotic mass surface along with ova. (d) Ferro-ferricyanide staining for tyrosine-based structures reveals an intensely stained vitellarium (arrow) and lighter staining in other areas of the mass likely due to the breakdown of the vitellarium. (a)–(d) Bars = 100 *μ*m.

Three histochemical procedures provided the most significant results with antennal gland tubule staining: AB2.5, PAS, and FF. The staining of acidic mucosubstances with AB2.5 revealed an interesting pattern in the antennal gland tubule cells, in which the stain was pronounced along the apical microvillar border and also along the basal border of the cells but not within the main soma of the cells (Figure 2(a)). The nuclei of antennal gland tubule cells were conspicuously clear with this stain. This staining pattern was present in cells touching the parasite as well as those distant from the worm, possibly indicating little effect of the parasite on the kidney tubules. The exception to this staining pattern applied to cells that were compact and flattened due to physical trauma. In flattened and squamous cells, staining differences between the soma and borders of the cells were not discernable. The PAS stain also revealed more intense staining of the brush border and basal side than within the cytoplasm of the tubule cells (Figures 2(b) and 3(c)). The AB2.5 and PAS stain both revealed areas where the brush border of the tubule cells had been scraped off by the worm though the tubule cells were intact. The third important histochemical result was the absence of a melanin reaction by the host, as revealed by the FF stain (Figure 2(d)). This test was important as decapods are known to produce melanin in their antennal glands as a result of parasitic infection.

### 3.3. Summary of* Alloglossidium renale* Histochemistry following Parasite Death (Figure 3, Table 4)

A necrotic* A. renale* was recovered and processed for a variety of stains (Table 4). This necrotic worm was a mass of unidentifiable material with ova and debris (Figure 3(a)). Hematoxylin stained the inner mass brown, though parasite nuclei and organs were not identifiable and the appearance of the mass was completely changed from that of the healthy parasite. Remnants of the tegument surrounded the mass and stained lightly. Damaged, squamous host tubule cells were fused to the remnants of the tegument. Tubule lumenal contents near the necrotic mass stained lightly.

The integrity of the parasite internal structure was unrecognizable with HH staining and this was also revealed with the histochemical tests. Specifically, there was a change in the parasite surface as AB2.5 staining no longer highlighted the surface of the worm, and a darkened staining of the outer necrotic mass with PAS and NS was present (Figures 3(b) and 3(c)). The PAS staining of the outer surface was present in the worm tegument and adherent host cells, and the NS staining of the outer surface was attributable to the adherent host cells. These changes along with the HH staining revealed a breakdown in structure. Host tissue that was not abraded or compressed revealed little change with the carbohydrate stains in that the brush border and basal layers of the host tubule cells were highlighted by AB2.5 and PAS.

Significantly, the FF stain revealed melanin or tyrosine products within the necrotic mass whose pattern differed from the intact mature worm. Ferro-ferricyanide stained the inner necrotic mass, necrotic tegument remnants, and cytoplasm green, while the vitellarium stained intensely green (Figure 3(d)). No staining with FF was attributable to the host tissue. Additionally, no structures were revealed with any stain that indicated a host reaction to worm even upon its death, and no structures were present to wall off or isolate the necrotic parasite with the exception of flattened host tubule cells affixed to the surface of the mass.

## 4. Discussion

This first report of* Alloglossidium's* histochemical nature has revealed a benign effect of the parasite on the host that suggests that this symbiotic association is well established and perhaps evolving toward commensalism. This study reports a positive ferro-ferricyanide reaction within the parasite while being alive and also in later stages of infection, when the trematode is necrotic, but not within the antennal gland tissue itself. Melanin is produced as a result of tissue damage in crustaceans and several species of shrimp produce a melanin reaction to damage or disease [15, 16]. A previous study of the trematode* Allocorrigia filiformis* in the antennal gland of the crayfish* Procambarus clarkii* recorded host hemocytes secreting melanin around ova in the interstitial space of the antennal gland [15]. Landers and Jones [8] reported no visible melanin associated with* A. renale* infections in grass shrimp, though traces of melanin may not have been observed because specific stains were not used on their plastic sections. Ferro-ferricyanide reacts strongly with melanin [12] and our results show that this reaction also occurs with the precursor of melanin production, tyrosine. Tyrosine is found in the vitellarium, as this organ produces not only yolk but also egg shell precursors which contain tyrosine as trematode egg shells are produced by quinone tanning [17]. Thus, we observed strong ferro-ferricyanide reactions within the vitellarium and to a lesser extent within the egg shells. The reaction observed in the necrotic mass was unexpected as was the negative reaction in the host. It is likely that the positive reaction throughout the necrotic parasite resulted from the atrophied vitellarium. The lack of a host response suggests that the host is well adapted to this parasitic infection.

The tegument of trematodes is typically covered with spines and contains secretory vesicles and granules [18, 19], and, in* A. renale*, the tegument aids in the destruction of host antennal gland tubules as the host tissue is compressed and abraded [8]. Carbohydrate staining of the tegument demonstrated that it is positive for acidic sulfated mucosubstances (AB1), acidic carboxylated mucosubstances (AB2.5), and neutral sugars (PAS), likely attributable to secretory vesicles. We observed AB2.5 positive material external to the worm and trapped within the suckers, but a uniform covering surrounding the parasite was not present. The question of a cyst wall or the production of an incomplete wall or envelope is important in the case of* A. renale*, as the parasite is progenetic, bypasses the metacercaria stage, and develops directly to an adult within the 2nd intermediate host (the definitive host in this life cycle). This species is reported to produce no metacercarial wall [8, 10] and that was explored in this study with histochemical staining. Earlier studies have demonstrated that metacercarial cysts may contain acid mucosubstances and proteins [20] and can be stained with alcian blue, PAS, ninhydrin, and stains specific for tyrosine [21]. This study did not reveal a metacercarial wall or any remnants of one surrounding the tegument.

Histochemical differences between healthy and necrotic trematodes were pronounced. No identifiable organs or cells except for evidence of an atrophied vitellarium were observed within the necrotic trematode. This outer layer of the remnant consisted of the worm tegument along with attached damaged host tubule cells. The host cells stained strongly with NS and HH. This walling off of the necrotic mass by squamous tubule cells appears to be the only response of the host to the infection. A similar host response was reported in a study of* Nanophyetus salmincola*, in which the host (salmon) formed a renal epithelial cell envelope around the metacercaria of the parasite [21].

Infections of* A. renale* caused changes in antennal gland tubule histology but only for those cells in direct contact with the parasite. The host tubule loss was attributed to feeding, abrasion, and compression from parasite growth, with compressed tubule cells always located in close proximity to the parasite's tegument. The compression of tubule cells caused obvious morphological changes to occur, in agreement with previous studies [8], though few histochemical changes were evident in antennal gland cells. No organ-wide effects were seen due to infection and no inflammatory reaction was observed as has been reported for other progenetic trematode kidney infections [22]. This reveals an overall tolerance for the infection that the remaining antennal gland tissue possessed. Host tubules are known to be functional even when most of the organ has been destroyed by the parasite, demonstrated by ova observed downstream from the parasite.

The relationship between the host and parasite is believed to be well established, with earlier studies of* A. renale* providing support for this interpretation [10]. Landers and Jones [8] showed no significant body length differences between infected and uninfected shrimp and showed that the parasite did not always destroy the host antennal gland before parasite death and necrosis. Also, that study and the current study reported functional tubules within infected antennal glands. The current study found no host response that limits parasite growth or development, suggesting that the antennal glands, which are used for reabsorption of glucose, excretion, and ion regulation [23], are not essential to the host. Several species of palaemonid shrimp use their gills for ion regulation and excretion [24, 25], and it is possible that the gills of* P. kadiakensis* are used to compensate for the loss of the antennal glands.

## 5. Conclusions

This first histochemical examination of* A. renale* and surrounding host tissue demonstrated no metacercarial wall or secretion to isolate the parasite from its host. Additionally, no differences in host tubule histology were found except for those cells in close proximity to the worm, and no host response was elicited from infection. Histochemical staining of the necrotic mass revealed the breakdown of the vitellarium within the worm but did not reveal other tyrosine-based structures surrounding the worm or within the tubules that would suggest a host melanin response. The only host response appeared to be a development of squamous cell envelope around the necrotic worm, which may be passive. These results support the interpretation that the* A. renale* relationship with its host is highly evolved and causes little harm to its host.

## Figures and Tables

**Table 1:** Stains and staining specificity.

Stain	Staining specificity	Controls: positive (P) and negative (N)
Alcian blue (pH 1.0) [AB1]	Strongly acidic (sulfated) mucosubstances stain blue	Fish intestinal submucosa (P)

Alcian blue (pH 2.5) [AB2.5]	Weakly acidic (carboxylated) mucosubstances stain blue	Fish intestinal submucosa (P) Shrimp hepatopancreas (P)

Ferro-ferricyanide [FF]	Melanin and tyrosine stain dark green/blue	Vitellarium (P)

Feulgen nuclear reaction [FL]	DNA stains red	Shrimp antennal gland nuclei (P) Schiff reagent without HCL treatment (N)

Harris hematoxylin [HH]	Nuclei stain dark purple and cytoplasm light brown	Shrimp antennal gland cells (P)

Ninhydrin Schiff [NS]	Amino acids stain pink or red	Shrimp muscle (P) Schiff reagent without ninhydrin treatment (N)

Osmium [OS]	Lipids stain black or olive	Oil droplets in cytoplasm (P)

Periodic acid-Schiff [PAS]	Neutral carbohydrates stain red	Fish intestinal goblet cells (P) Schiff reagent without PA (N)

Toluidine blue [TB]	Nuclei stain dark blue, acidic mucin and cytoplasm stain light blue, pink, or violet	Shrimp muscle and antennal gland nuclei (P)

Ward's protein stain [WPS]	Amino acids stain blue	Shrimp muscle (P)

**Table 2:** *Alloglossidium renale* adult worm *in situ* staining results.

Stain	Tegument	Parenchyma	Vitellarium	Intestinal lining	Ova
Alcian blue (pH 1.0)	+++ outer tegument	−	−	+	−

Alcian blue (pH 2.5)	+++ outer tegument	−	−	++	−

Ferro-ferricyanide	−	−	+++	−	+

Feulgen nuclear reaction	++ inner tegument	+	++	−	++

Harris hematoxylin	+ outer tegument light brown cytoplasm + inner tegument dark purple nuclei	++ light brown cytoplasm + dark purple nuclei	+++ dark purple nuclei	+ cytoplasm ++ dark purple nuclei	+ cytoplasm ++ dark purple nuclei

Ninhydrin-Schiff	++	+ light	+++	++	+++

Osmium	−	++ random oil droplets	+	++	−

Periodic acid-Schiff	+ outer tegument ++ inner tegument	+	+/−	++	++

Toluidine blue	+ outer tegument light blue ++ inner tegument dark blue (Nuclei)	+ purple nuclei + pink acidic structures	+++ dark blue nuclei	+ dark blue nuclei ++ light blue acidic structures	+++ dark blue nuclei

Ward's protein stain	++	+	++/−	++	++

**Table 3:** *Palaemonetes kadiakensis* tissue staining table.

Stain	Tubule cell apical microvilli	Tubule cell basal border	Tubule cell nuclei	Tubule cell cytoplasm	Lumen of tubule	Interstitial Space	Damaged tubule cells
Alcian blue pH 1.0	−	−	−	−	−	−	−

Alcian blue pH 2.5	++	+	−	−	−	−	+

Ferro-ferricyanide	−	−	−	−	−	−	−

Feulgen stain	−	−	+++	−	−	−	+++

Harris hematoxylin	+	++	+++	+	+	−	+++ nuclei ++ cytoplasm

Ninhydrin-Schiff	+	+	++	+	−	−	++

Osmium tetroxide	−	−	−	−	−	−	−

Periodic acid-Schiff	++	++	−	+/−	+	−	+

Toluidine blue	−	+ light blue	++ dark blue	+ light blue	+	−	+++ dark blue nuclei + light blue acidic structures

Ward's protein stain	+	+	−	+	+	−	+

**Table 4:** Necrotic *Alloglossidium renale *tissue staining table.

Stain	Inner necrotic mass	Necrotic mass surface	Lumen of host antennal gland tubules
Alcian blue pH 2.5	−	++ sporadic	−

Ferro-ferricyanide	+++ vitellarium	++ tegument	−

Harris hematoxylin	++ brown	+++ brown host cells and tegument purple host nuclei	+ light brown

Ninhydrin-Schiff	+ sporadic	++ host cells	+

Osmium tetroxide	+	−	−

Periodic acid-Schiff	++	++	+
